# Covalently modified carboxyl side chains on cell surface leads to a novel method toward topology analysis of transmembrane proteins

**DOI:** 10.1038/s41598-019-52188-4

**Published:** 2019-10-31

**Authors:** Anna Müller, Tamás Langó, Lilla Turiák, András Ács, György Várady, Nóra Kucsma, László Drahos, Gábor E. Tusnády

**Affiliations:** 1 Institute of Enzymology, RCNS, Hungarian Academy of Sciences, Magyar Tudósok krt 2, Budapest, H-1117 Hungary; 2 Institute of Organic Chemistry, RCNS, Hungarian Academy of Sciences, Magyar Tudósok krt 2, Budapest, H-1117 Hungary

**Keywords:** Biological techniques, Computational biology and bioinformatics, Structural biology

## Abstract

The research on transmembrane proteins (TMPs) is quite widespread due to their biological importance. Unfortunately, only a little amount of structural data is available of TMPs. Since technical difficulties arise during their high-resolution structure determination, bioinformatics and other experimental approaches are widely used to characterize their low-resolution structure, namely topology. Experimental and computational methods alone are still limited to determine TMP topology, but their combination becomes significant for the production of reliable structural data. By applying amino acid specific membrane-impermeable labelling agents, it is possible to identify the accessible surface of TMPs. Depending on the residue-specific modifications, new extracellular topology data is gathered, allowing the identification of more extracellular segments for TMPs. A new method has been developed for the experimental analysis of TMPs: covalent modification of the carboxyl groups on the accessible cell surface, followed by the isolation and digestion of these proteins. The labelled peptide fragments and their exact modification sites are identified by nanoLC-MS/MS. The determined peptides are mapped to the primary sequences of TMPs and the labelled sites are utilised as extracellular constraints in topology predictions that contribute to the refined low-resolution structure data of these proteins.

## Introduction

Transmembrane proteins (TMPs) having at least one transmembrane segment, are located in the phospholipid bilayers of the cells. TMPs are crucial in several biological pathways such as the extracellular or intracellular transporting or signalling^1–3^. Approximately 55% of the drugs authorised by the Food and Drug Administration interact with TMPs^4^ so the 3D structure of these proteins could be necessary for computational drug design. While around 20–30% of the open reading frames of the human genome code TMPs^5–7^, only 2% of the previously solved protein structures belong to TMPs^8–11^.

The determination of high resolution TMP structures is still complicated, so topology became a tool for representing low resolution data instead of the exact 3D structure^12,13^. Topology is the most frequently used representation of a TMP structure, defining the relative location and orientation of the transmembrane (TM) regions, the extracellular and intracellular loops to the membrane itself and the number of the TM regions.

Topology predictions remain a necessary method for the structure analysis of TMP structures^14^. Earlier predictions are built upon the secondary structure based on the chemical characteristic of the amino acids, searching for hydrophobic regions^15^. As bioinformatics developed, machine learning also appeared in the research toward topology of TMPs^13,16–19^. As supervised machine learning methods always utilises a learning set of data, we cannot avoid miscalculations on unknown protein families^20,21^. Machine learning itself has increased the accuracy of the predictions up to a point but with utilising the experimental topology data, the accuracy of the predictions increases remarkably^12,13,22^.

There are plenty of examples how topological data of TMPs can be experimentally determined with or without the modification of the coding sequence of the protein of interest. Several experimental methods were developed for the analysis of the topology of modified proteins such as protein fusion, epitope insertions, glycosylation motif insertion, C- or N-terminal tagging, single amino acid mutagenesis screening combined with different crosslinking agents^23–28^, however these procedures are time-consuming and the interpretation of the results are sometimes not straightforward^24^. Moreover, the function of the modified protein is sometimes different compared to the wild-type protein, for example the function of the modified human folate transporter 1 (SLC19A1, S19A1_HUMAN) changed in certain cases^29^. The *in vivo* or *in vitro* translation of the modified TMPs might be extremely difficult^30^ so we mainly focus on experiments without modification of the coding sequence. Among others, examining the extracellular region of the native protein through glycosylation sites contributes to the known topology of TMPs^31^, especially by creating high-throughput glycosylation data banks (e.g. Cell Surface Protein Atlas)^32^. Partial proteolysis also provides small resolution data based on the known cleavage sites of the applied proteases and these sites can be detected even by the fragments of the examined proteins via SDS-PAGE^33,34^. The locations of endogenous epitopes are also able to provide low-resolution topology data of TMPs according to the applied antibody as for example in the case of wheat Aluminum-activated malate transporter 1 (ALMT1_WHEAT)^35^.

In particular, the chemical modifications on the reactive side chains of accessible amino acids make the examination of their relative location to the membrane in a native TMP^36^ possible. There are plenty of labelling agents available on the market and most of them are specific for several functional groups. Many crosslinking reactions have already provided distance constraints for the 3D structure determination of proteins based on the length of these spacer arms^37^. Besides intramolecular interactions, intermolecular crosslinking is also available this way^38^. In certain cases, by applying membrane-impermeable agents, it is also possible to provide topology information on TMPs^27,32,39^.

The most popular amino acids for these covalent modifications are cysteins and lysines because sulphydrils and primary amines are reactive enough for a one-step modification by an appropriate chemical agent^40,41^. For the modification of sulphydrils, maleimides or pyridyl disulfides are mostly applied and for the primary amines, imido esters or N-hydroxysuccinimide esters are typically utilised^42^. Beside the most reactive side chains, there are two more amino acids whose reactivity is quite satisfying so it is also possible to modify the side chains of aspartic and glutamic acids^43,44^ although these carboxyl groups are mostly modified in a two-step reaction^45^. As during the artificial peptide synthesis, the carboxyl group has to be activated before adding the free primary amine to the reaction. Using carbodiimides combined with succinimides in an acidic environment is a popular method for the activation step^46,47^, which is followed by the labelling step. The formation of the amide group can only occur at a slightly alkaline pH because the amine group has to be deprotonated (Supplementary Fig. 1). On the other hand, the stability of the activated carboxyl groups incredibly decreases at higher pH^48^.

Recently, we have developed an experimental method for the determination of extracellular lysine side chains of TMPs to provide topology data of them that can be utilised by the CCTOP prediction algorithm in order to achieve better prediction accuracies^39^. The experiments of the workflow are based on a method that allows the high throughput and accurate identification of extracellular lysine side chains that were modified with a membrane-impermeable labelling agent. This way, partial labelling of TMPs generated sufficient constraints to significantly increase the reliability and accuracy of topology predictions^39^.

On the other hand, labelling the extracellular lysine side chains also has disadvantages. In order to produce the most detectable fragments for the nano liquid chromatography tandem-mass spectrometry (nanoLC-MS/MS), tryptic digestion is applied in most of the proteomic experiments^49–51^. Unfortunately, trypsin enzyme does not recognize the covalently modified lysine side chains^50,52^ so a great amount of missed cleavages appear. Furthermore, peptides containing the dedicated covalent modifications cannot be straightforwardly sequenced this way due to their length and the fact that the labelled lysine side chains do not carry the positive charge which is crucial for MS/MS fragmentation^39^. To summarise, the digestion and the labelling are limiting each other so a need arose for a different labelling method.

The aim of this study was to provide an alternative labelling method to avoid the above described difficulties for generating further experimental topology data for the CCTOP algorithm and an accurate labelling method where the targeted amino acid side chains are modified only on one side of the membrane. Here, we targeted the extracellular carboxyl side chains of the TMPs by covalently modifying them with membrane-impermeable activating agents combined with a biotinylating reagent. To the best of our knowledge, only a few publications are connected with the modification of these amino acids^53,54^ so the optimisation of these experiments seems to be a novel project for the development of the research toward the better understanding of the topology of TMPs.

## Results

### Detection of the covalent modification on model protein

**Figure 1:**
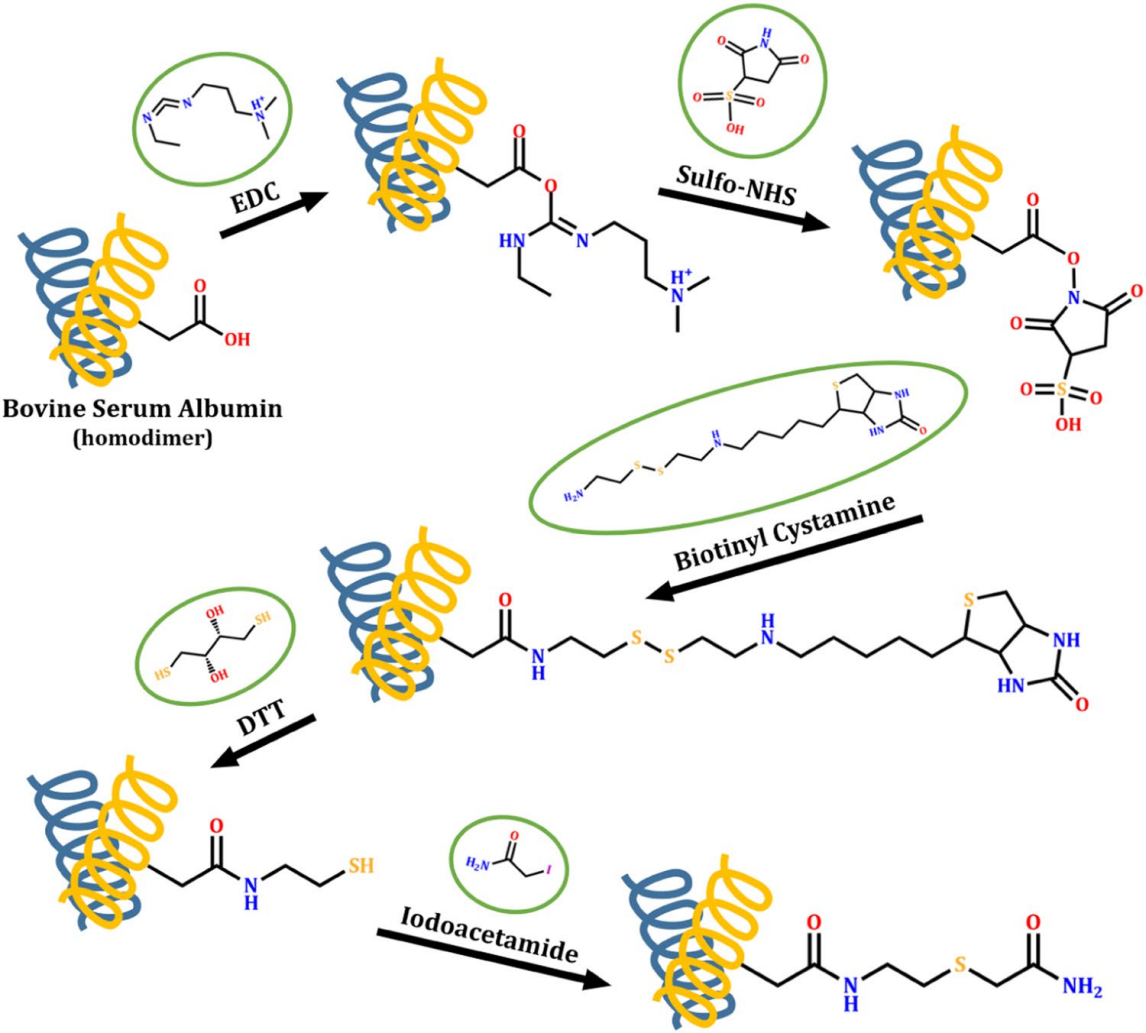
The reaction flow of the applied labelling method. The carboxyl amino acid side chains of Bovine Serum Albumin model protein were activated by EDC and Sulfo-NHS, and then biotinylated by Biotinyl Cystamine. During the nanoLC-MS/MS sample preparation, the disulphide bridges were cleaved by DTT and alkylated by Iodoacetamide reagents.

**Figure 2:**
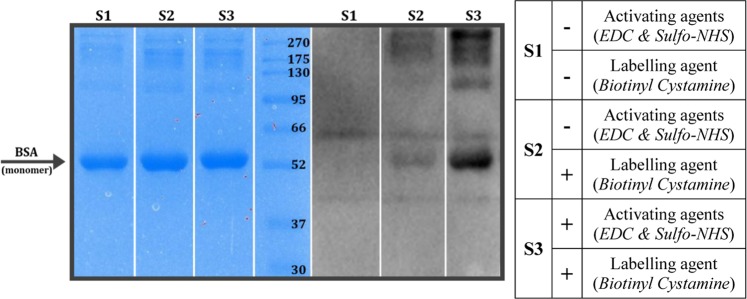
SDA-PAGE and Western Blot analysis of the biotinylated BSA model protein. The protein successfully bound the biotin-residues in both experiments when the labelling agent was applied but the activation step resulted in a higher biotinylated BSA yield. Images were captured by a Bio-Rad ChemiDoc XRS+ Imaging system. The full-length images are presented in Supplementary Fig. 2.

We have successfully modified the carboxyl side chains of a model protein (Bovine Serum Albumin, BSA) with a biotin-containing agent (Fig. 1). The semi-quantitative results prove the importance of the activation step before biotinylation. Although we have observed the presence of the modification in the non-activated sample, the reaction was much more effective on the activated carboxyl groups (Fig. 2). Since BSA usually forms homodimers and we applied 98% pure BSA, some nonspecific bands also appeared.

The applied labelling and isolation steps lead to a known covalent modification on the carboxyl side chains. The successfully alkylated groups are modified by +116.040819 Da while the non-alkylated groups by +59.019355 Da. The nanoLC-MS/MS method followed by data analysis allowed the detection of these modifications (Supplementary Tables 5 and 6).

**Figure 3:**
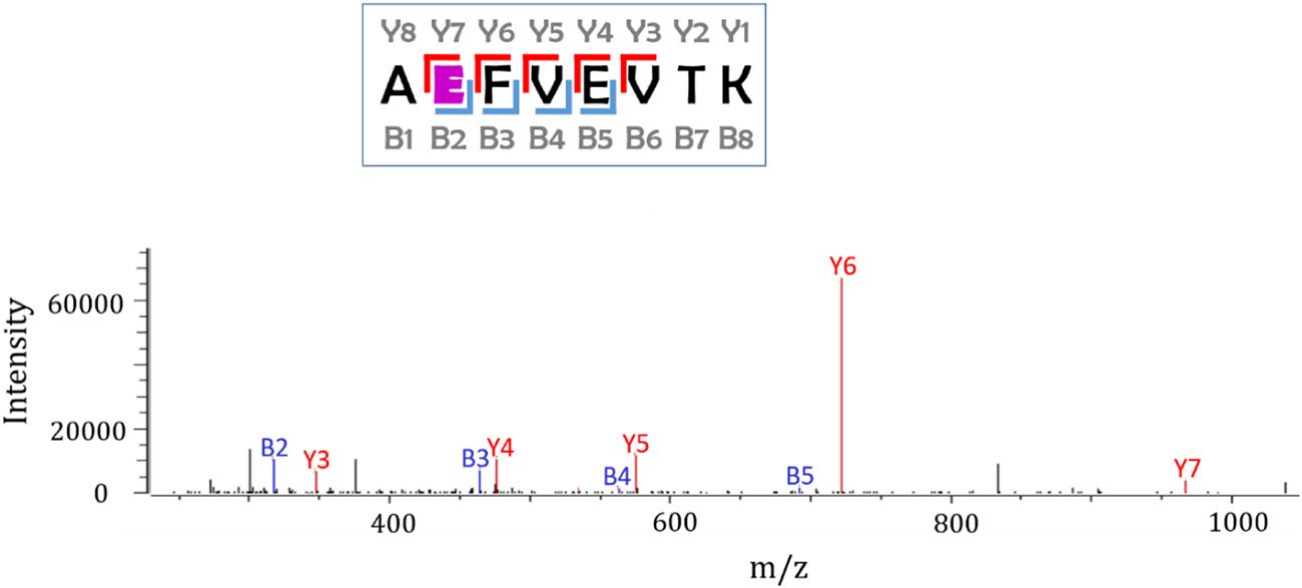
The Intensities of the fragments of a successfully modified BSA peptide. During the applied nanoLC-MS/MS analysis, we were looking for the known fix covalent modifications on the carboxyl amino acid side chains of BSA model protein. Based on peptide sequencing, here we present the +116 Da modification on the highlighted aspartic acid (E). The image was created by Byonic 2.15.7 (Protein Metrics Inc., Cupertino, CA, USA)^67^.

The presence of the Y_7_, B_2_, B_3_, B_4_ and B_5_ fragments prove the successful modification of the highlighted aspartic acid (E_226_) by +116 Da (Fig. 3). Altogether 143 modified BSA peptides resulted 29 modified aspartic or glutamic acid positions in the native model protein. The list of the labelled BSA peptides is shown in Supplementary Table 1.

### FACS measurement

**Figure 4:**
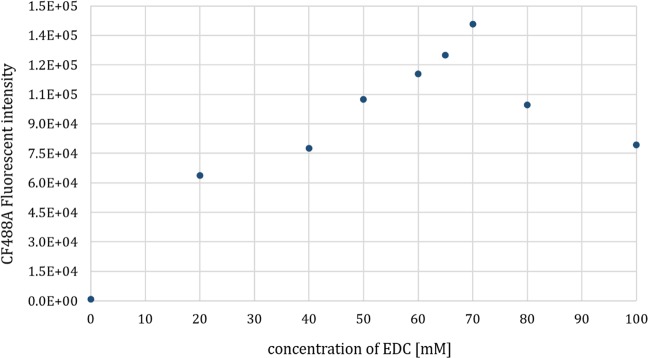
The effect of different activating agent concentrations on cell surface biotinylation. EDC and Sulfo-NHS were applied in a 1:2 molar ratio. According to the fluorescent intensities emitted by CF488A anti-biotin antibody, we found that cell surface biotinylation did not increase when applying the activating agents over the concentrations 70 mM EDC and 140 mM Sulfo-NHS.

While extending the biotinylation method for labelling the extracellular carboxyl side chains of TMPs on the surface of living cells, it was essential to detect the effect of the reaction mixtures on living cells. We applied different concentrations of the activating agents then measured biotinylation and cell death by flow cytometry. Both cell death by Propidium Iodide uptake and successful surface labelling by CF488A fluorescent anti-biotin antibody were measured. First, we examined a wide range of EDC/Sulfo-NHS concentrations and we found a maximal fluorescent intensity of the applied biotin-binding antibody at 70 mM EDC and 140 mM Sulfo-NHS concentrations (Fig. 4 and Supplementary Fig. 4).

According to the Propidium Iodide uptake, the rate of cell death grows by the increase of the concentration of the activating agents but more than 99% of the examined cells were alive even at the highest concentration (Supplementary Fig. 4).

### Confocal microscopy

**Figure 5:**
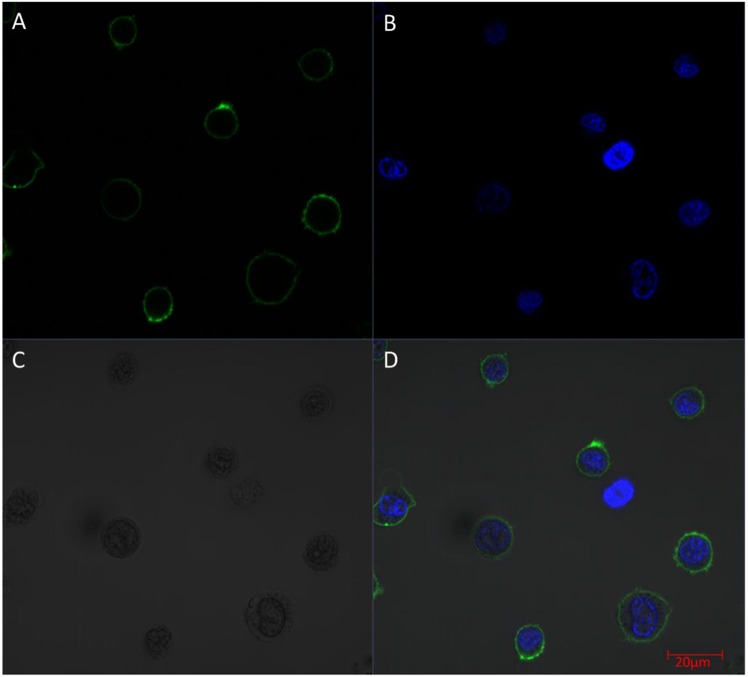
Examining the surface biotinylation of the cells by applying the observed maximal labelling concentrations. The fluorescent signal of the applied dyes and the HL60 cells were detected by confocal microscopy (A: Alexa Fluor 488 conjugated anti-biotin antibody fluorescence, B: Hoechst 33342 DNA dye fluorescence; C: Differential Interference Contrast; D: Merged picture). Scale bar: 20 µm. The images were created by Zeiss ZEN lite software (Carl Zeiss, Oberkochen, Germany).

The suspected maximal intensity of cell surface labelling at 70 mM EDC and 140 mM Sulfo-NHS was further examined by confocal microscopy. The CF488A fluorescent labelling of the cell surface was homogenous and there was no signal in the cytoplasm which indicated that the applied reagents in the above described parameters do not impair the integrity of the cells (Fig. 5). The results of the control experiments are presented in Supplementary Fig. 5.

### Enrichment of extracellular protein segments

Labelled cells were solubilised, which was followed by the digestion of the membrane preparations. We enriched the biotin-containing peptides via affinity chromatography. The quality of the peptides was tested by blotting the samples onto a PVDF membrane.

The amount of the used neutravidin beads did not restrict the isolation of the total amount of the biotinylated peptides. Comparing the biotin content of the solutions that were taken before and after affinity column, it was clear that all the biotinylated components stayed on the neutravidin beads which means that we used sufficient amount of the neutravidin beads (Supplementary Fig. 6).

Covalently labelled peptides were eluted by DTT reducing agent from affinity column then we applied iodoacetamide alkylating agent in order to avoid the aggregation of peptides through disulphide bridges. The method resulted in the covalent modification of extracellular carboxyl groups by +116.040819 or +59.019355 Da.

### Identification of the labelled peptides by tandem mass spectrometry and validation of the experimental results

After setting the labelling parameters on BSA model protein and finding the best concentrations for the labelling agents by FACS and confocal microscopy that resulted in high fluorescence intensity on cell surface but left cells intact, we labelled human HL60 cells. The biotinylated cells were lysed, and the membrane preparations were solubilized and digested. Finally, the labelled peptides were isolated on neutravidin column. The list of the tandem mass spectrometry identified peptides carrying the modified carboxyl groups is shown in Supplementary Table 2 (raw ms data tab).

Altogether 1096 peptides containing covalently modified aspartic acid or glutamic acid were detected by nanoLC-MS/MS. The labelled peptides of HL60 cell line from the different Byonic searches were linked to individual proteins, and the sites of modifications in unique proteins were counted. We account only those aspartic or glutamic acids that were identified in at least three independent labelling experiments. This resulted in 135 positions in 38 TMPs (Supplementary Table 2, re-mapped peptides tab).

## Discussion

Labelling the extracellular amino acid carboxyl side chains of TMPs in order to generate applicable data for their more accurate topology prediction and structure modelling is a novel tool. We utilised two membrane-impermeable activating agents and one biotinylating reagent in a two-step reaction. At first, the reaction was verified on a single protein (BSA) then extended to the surface of living cells. Both flow cytometry and confocal microscopy measurements confirmed the integrity of the cells. During the experiments, 135 new topological positions were identified for 38 TMPs in HL60 cell line by 16 nanoLC-MS/MS runs and from BSA digestions, 29 amino acid carboxyl side chains were detected from the accessible surface of the protein.

The majority of the identified TMPs (34 out of 38 labelled TMPs) has only one TM segment and a large extracellular domain. 30 of the 34 proteins are Single-pass type I proteins and the other 4 are Single-pass type II proteins. Their extracellular domain contains many glutamic and aspartic acids that are available for the chemical agents during the cell surface labelling procedure. The existing topology predictions are already accurate for these proteins due to this low structural diversity and the number of already existing experimental results (such as signal peptides, labelled lysine residues or identified glycosylation sites)^32,39,55^.

On the other hand, our protocol was also able to label multi-pass TMPs. There is no surprise that the predictions of multi-pass TMPs are often controversial because membrane-embedded parts are more complex, therefore re-entrant loops and interfacial helixes also appear, which could sometimes also mislead the prediction algorithm.

According to the HTP database, the predictions of the two TM-segment Cell cycle control protein 50A (CC50A_HUMAN) are quite synchronous. Unfortunately, there are only a few experimental positions available that could verify these topology predictions^31,56,57^ (for details see data in HTP database; HTP id: 002623). Here, we successfully labelled E_224_ aspartic acid that is localised between two transmembrane segments of this protein. The extracellular location of their interconnecting loop has already been confirmed by three independent experiments. Thus, previous experimentally determined extracellular data and our labelled position validate each other.

Leukocyte surface antigen CD47 protein (CD47_HUMAN) is consistently predicted to have five TM segments. On the other hand, the extracellular and intracellular segments are controversial based on existing predictions as shown in Supplementary Fig. 7. Previous experiments have already provided some extracellular positions in the first extracellular domain on the protein^31,56,58^ so the extracellular location of the labelled E_122_ aspartic acid is verified based on other experiments (HTP id: 002114). Using all the available information contributes to a more accurate topology prediction of this protein.

In case of the Neutral amino acid transporter B(0) protein (AAAT_HUMAN), different topology prediction algorithms provided various topology for this transporter (HTP id: 001260). Even the number of predicted membrane segments is different in some protein regions by different predictions. Our carboxyl-labelling method identified three different positions that prove the extracellular location of a loop region of the protein, which is consistent with the previously determined N-linked glycosylation site from this part^56^. Additionally, the 3D structure of this protein was solved by cryo-electron microscopy^59^ lately so the results of our experiments were also examined by using this data. The structure of this protein (PDB code: 6GCT) suggested that all three labelled positions are localised in the extracellular region (Supplementary Fig. 8) so the correctness of our data was confirmed for the second time regarding this protein.

Considering Equilibrative nucleoside transporter 1 (S29A1_HUMAN) protein, our experiments resulted in one labelled small extracellular loop, where the modified E_325_ amino acid is relatively close to the membrane region. This way, we were also able to label small extracellular loops close to the membrane despite of the possible spherical effects and to the best of our knowledge, this extracellular segment has not been experimentally verified yet (HTP id: 002245). Furthermore, this modified amino acid confirms that it is also possible to identify labelled positions from smaller extracellular loop regions as opposed to previous examples.

32 out of the 38 identified TMPs have been investigated experimentally so far. According to these experiments, all the labelled carboxyl groups are localised in the extra-cytosolic region. Clustering proteins that contain at least one peptide with modified carboxyl group and identified at least three times independently resulted in 95 TMPs altogether.

Today, many of the available high-throughput method identified glycosylation sites are based on the Cell Surface Protein Atlas (CSPA) where extracellular glycosylation positions were determined by LC-MS/MS^32^. The CSPA dataset also contains information about HL60 cell line that was used in our experiments too. 194 proteins were identified totally from HL60 cell line by their cell surface capture technology. According to the CCTOP algorithm, 172 out of them are TMPs. Considering all these TMPs, only 21 of our carboxyl-labelled TMPs are already shown in CSPA. The extracellular regions of the 17 new TMPs contain much more aspartic and glutamic acids than glycosylation sites so our labelling method might have detected them easier.

Additionally, the +0.984 Da modification resulted on the asparagine amino acids by deglycosylation with PNGase F enzyme sometimes cannot be unambiguously detected by LC-MS/MS because the deamidation can also spontaneously occur on asparagine residues^60^. This is the reason why the identified extracellular positions are less reliable in CSPA compared to our carboxyl-labelling experiment.

Regarding the already existing topology predictions and experiments, all of the labelled carboxyl groups are located in the extracellular region, therefore the data produced by carboxyl group labelling is 100% accurate. Supplementary Table 3 contains the predicted topology of the modified TMPs including the modified aspartic and glutamic acids from our experiments and also the already existing other experimental results. Interestingly, the rate of labelled aspartic and glutamic acids is different. Glutamic acid side chains were modified around twice as often as aspartic acid side chains because the longer amino acid side chains could be more accessible to the applied chemical agents.

Characterising the Custom modifications of +59.019355 and +116.040819 Da, we have to evaluate that while 2% of the modified HL60 peptides contained the non-alkylated +59.019355 Da modification, applying the “labelled position per 3 peptide” filter, 100% of the analytically accepted modifications on TMPs are +116.040819 Da.

Here, we would like to take the opportunity to compare the labelling yield of the carboxyl side chain targeted method to the lysine-labelling procedure. Our previous publication was built upon the analysis of three cell lines including HL60^39^. In case of this acute myeloid leukemia cell line, 371 positions were detected in 114 TMPs via the lysine-labelling protocol by nanoLC-MS/MS on a Bruker Maxis II ETD Q-TOF mass spectrometer in 12 measurements. There are 13 TMPs that were detected by both protocols and the carboxyl-labelling method resulted in 25 new TMPs. Although 24 out of these 25 TMPs show homology with the previously lysine-detected TMPs, Equilibrative nucleoside transporter 1 (S29A1_HUMAN) protein was only captured by the carboxyl-labelling method. According to CCTOP algorithm, this protein contains 3 extracellular lysine residues beside 11 aspartic or glutamic acids. Even if there is a quite reasonable difference between the TMP-yield of the two experimental protocols, less false labelling appeared in our newly presented carboxyl targeted experiments due to the selective labelling and the several washing steps.

Beside 38 TMPs, we also labelled 5 non-TM proteins. According to the Peripheral Membrane Protein database^61^ that contains proteins attached to the plasma membrane, 2 out of these 5 proteins are in the database, furthermore they are located on the extracellular region of the cell (PDIA1_HUMAN & B2MG_HUMAN) so our protocol made it possible to label peripheral membrane proteins on the cell surface too.

To summarise the advance of the optimised carboxyl-labelling experiments described here, we can state that providing topology data for TMPs require 3–4 weeks which is a shorter period than by previously existing techniques that mainly characterised a single protein or a smaller protein family. Moreover, culturing parallel cell lines from different origin could contribute to topology data of many hundreds of TMPs which could exponentially increase the number of known topologies.

Beside labelling and isolating living cell surface, the covalent modification of the carboxyl side chains has other possibilities. Among others, our developed labelling protocol will make protein carboxyl residues available targets for other conjugation techniques. For example, antibody-drug conjugations based on antibody-antigen interactions also require free functional groups on the heavy chain of the antibody^62^. In this field of study, cysteines are mostly targeted through their sulphydril-reactions, but it also has several disadvantages. The native protein structure is quite sensitive and the complex dissociates easily because the heavy and light chains of the antibody bind each other through disulphide brides of the cysteine residues^63^. The modification of amino acid residues in novel proteins usually target cysteine or lysine residues^64^. Here, we prove that aspartic and glutamic acids could also become a satisfying target for several conjugation experiments.

We would like to highlight the possibility that the carboxyl-labelling protocol will soon become a tool for cell surface isolation either used individually or in combination with the lysine-labelling method. Although carboxyl-activating reagents were previously used for other techniques^36^, it is clear that protonated and covalently modified lysine side chains do not bind the activated carboxyl groups. The applied washing steps and pH values allowed our selective conjugation reaction. In consideration of the above described advantages, this optimised carboxyl-labelling protocol could be the basis for a new cell surface-isolation kit in the near future.

## Methods

For the unity of the text, the details of the applied materials are indicated in Supplementary Table 7 and the applied instruments and machines in Supplementary Table 8.

### Labelling the carboxyl groups of a model protein

In preliminary experiments, we have applied a model serum protein, Bovine Serum Albumin (100 µg BSA). At first, the carboxyl groups were modified either in a one-step or in a two-step reaction. The one-step reaction contained only biotinylation by 1 mM Biotinyl Cystamine. During the two-step reaction, 3 mM 1-ethyl-3-(3-dimethylaminopropyl) carbodiimide hydrochloride (EDC) and 6 mM N-hydroxysulfosuccinimide (Sulfo-NHS) were applied in acidic environment (200 mM MES, pH = 5.0) for the activation followed by the biotinylation step with 1 mM Biotinyl Cystamine.

### Detection of the covalent modification on model protein

For detecting the labelling efficiencies, we applied SDS-PAGE and Western Blot. The model protein samples (5 μg) were loaded on a 12% SDS-PAGE and stained with Coomassie Brilliant Blue. For the semi-quantitative Western Blot, HRP-conjugated Streptavidin and Immobilon Western Chemiluminescent HRP Substrate were applied.

The biotinylated protein samples (50 µg BSA) were incubated at 37 °C for 16 hours with MS-grade trypsin in a 1:100 (w/w) protease:protein mass ratio in the presence of 0.1% (w/v) Rapigest surfactant. The digestion was inactivated by heat (95 °C for 10 min) and 1 mM TLCK inhibitor (room temperature for 30 min). For the cleavage of the disulphide bound, the samples were incubated with 50 mM NH_4_HCO_3_ (pH = 8.0) buffer containing 10 mM 1,4-dithiothreitol (DTT) for 1 hour at 37 °C. In order to avoid further disulphide-bridge formation, free sulfhydryls were alkylated with 22 mM iodoacetamide.

The peptides were then purified on C18 spin columns and diluted in 20 µl loading buffer containing 2% acetonitrile and 0,1% formic acid. 6 µl of the solution was injected onto the nanoLC-MS/MS.

### Cell cultures and isolation

HL60 – which is an acute promyelocytic leukemia cell line - cells were obtained from American Type Culture Collection and were cultured in RPMI supplemented with 50 µg/ml Penicillin-Streptomycin and 10% Fetal Bovine Serum (FBS) in a humidified 37°C incubator with 5% CO_2_ atmosphere. All media were sterile filtered by bottle-top vacuum filter systems (0.2 µm pore size). HL60 cells were collected by centrifugation at 300 g for 5 minutes at 4 °C and washed with PBS (pH = 7.3; 137 mM NaCl, 2.7 mM KCl, 10 mM Na_2_HPO_4_ and 1.8 mM KH_2_PO_4_) three times. During the last washing step, we applied 4 mM iodoacetamide alkylating agent in order to avoid the production of “piggyback” peptides on free sulfhydryl groups (“piggyback” peptides are cysteine containing peptides that can bind to each other through disulphide-bridge)^65^. For MS analysis 10^8^ HL60 cells were used in each experiment.

### Labelling the carboxyl groups on the surface of living cells

For each sample, we used 2*10^6^ HL60 cells that were cultured and isolated as previously described. For the activation step, we applied the agents in a 1:2 molar ratio in a range of 0–150 mM for EDC and 0–300 mM for Sulfo-NHS in an activating buffer (pH = 5; 100 mM MES, 150 mM NaCl) at 4 °C for 15 minutes. It was followed by a washing step, where the activating buffer was applied at a higher pH (pH = 6.5; 100 mM MES 150 mM NaCl). The biotinylation step was performed using 1 mM Biotinyl Cystamine in PBS (pH = 8.0). The labelling reaction was stopped by adding 100 mM glycine (in PBS, pH = 7.3). The labelling efficiency was first detected by Dot Blot technique (Supplementary Fig. 3).

### FACS measurement

The labelled cells were washed with PBS (pH = 7.3) and incubated with PBS containing 2% (w/v %) BSA before applying CF488A fluorescent anti-biotin antibody (200x diluted) and Propidium Iodide DNA-dye (1000x diluted). The measurements were conducted by FACS Attune Acoustic Focusing Cytometer.

### Confocal microscopy

For the maximal fluorescence anti-biotin antibody intensity, we applied 70 mM EDC and 140 mM Sulfo-NHS during the activation step. Each sample was prepared as described in the flow cytometry protocol except for the DNA dying step. Instead of Propidium Iodide, Hoechst 33342 DNA-dye (10000x diluted) was applied. The samples were measured by Zeiss LSCM 710 Confocal Microscope (objective: 63x NA = 1.4 Plan Apo).

### Preparation of the cells for MS analysis

For mass spectrometry analysis, cell surface biotinylation was performed using the optimal 70 mM EDC and 140 mM Sulfo-NHS concentrations under the same activation conditions as mentioned above. The washing step, biotinylation and quenching were applied as previously described. Here, none of the fluorescent antibodies were utilised.

### Membrane preparation

We used the protocol described in our previous work (Lango *et al*.)^39^ with a few modifications. Shortly, a hypotonic lysis buffer (pH = 7.4, supplemented with 10 mM iodoacetamide) was applied for the lysis of the carboxyl-labelled HL60 cells a 4 °C for 10 minutes. Then the cells were disrupted by micro-pestle 40 times and we passed the samples 20 times through a needle (26 gauge). Cell lysate was centrifuged at 1700 g for 10 minutes at 4 °C for separating the cell debris and the nuclei. The supernatant was further centrifuged at 40000 rpm (in a Beckmann 70.1 Ti rotor) for 1 hour at 4 °C (by a Beckman Ultracentrifuge) for collecting biotinylated membrane fraction. Pellets were purified with washing buffer (pH = 7.7) and were centrifuged at 40000 rpm for a further 1 hour at 4 °C then resuspended in the washing buffer. We applied the method of Lowry *et al*.^66^ for the determination of the protein concentration in the membrane fraction using a standard stock solution of BSA.

### Membrane protein solubilisation and digestion

The solubilisation and digestion of the membrane proteins were similar to Lango *et al*.^39^. We applied a slightly alkaline buffer (100 mM NH_4_HCO_3_; pH = 8.0) supplemented with 0.1% (w/v) Rapigest surfactant and 1.2 mM iodoacetamide. The solution also contained 1.2 mM 2,2′ -thiodiethanol in order to prevent overalkylation of proteins and peptides during the digestion process. We also applied sonication to assist the solubilisation before incubating the samples for 30 minutes on ice. The suspension was treated with 500 units of PNGaseF and 60 units of α2-3,6,8,9 Neuraminidase A for 2 hours at 37 °C before adding trypsin in a 1:100 (w/w) protease:protein mass ratio. The samples were incubated at 37 °C for 16 hours then heat inactivation (at 95 °C for 10 min) was applied, finally 1 mM TLCK trypsin inhibitor was added to the solution for stopping the enzymatic digestion.

### Biotinylated peptide isolation

For the precipitation of the biotinylated peptides, high capacity neutravidin agarose resin was used as described in Lango *et al*.^39^. First, we monitored the binding capacity of the neutravidin columns by Dot Blot technique in case of the carboxyl-labelled samples (Supplementary Fig. 3). The biotinylated components of the solution were isolated on equilibrated neutravidin agarose resin (150 μl, 1 hour, room temperature). In order to reduce the number of nonspecific peptides or contaminants, we washed the columns extensively: 3 ml of each buffer was applied: first 50 mM NH_4_HCO_3_ (pH = 8.0), then 5 M NaCl in PBS, followed by 50 mM NH_4_HCO_3_ (pH = 8.0), 100 mM NaHCO3 (pH = 10.0) and hot (60 °C) 50 mM NH_4_HCO_3_ (pH = 8.0). Before the last washing step, we transferred the agarose resin into a new spin column. 50 mM NH_4_HCO_3_ (pH = 8.0) buffer supplemented with 10 mM DTT was applied for the elution of the biotinylated peptides from the immobilised neutravidin column. The samples were incubated for 1 hour at 37 °C before adding 22 mM iodoacetamide alkylating agent for preventing the formation of further disulphide bonds between the free sulfhydryl groups.

### Mass spectrometry analysis and peptide identification

The desalted samples were analysed by nanoLC-MS/MS similarly to Lango *et al*.^39^. The mass spectrometer was a Bruker Maxis II ETD Q-TOF, the ionization source was a CaptiveSpray nanoBooster source connected to a nanoLC (Dionex Ultimate 3000 NanoLC System). For peptide trapping, an Acclaim PepMap100 C18 Nano-Trap column (5 μm, 100 Å, 100 μm × 20 mm) was applied before separating them online by a Waters Acquity UPLC M-Class Peptide BEH C18 column (25 cm, 1.7 μm particle size). Components of the samples were analysed during 90 min gradient elution (4–50% eluent made of acetonitrile and 0.1% formic acid). The time of the MS measurements was fixed in a cycle of 2.5 sec and for generating the MS spectra, 3 Hz was set in the 150–2200 m/z mass range. For abundant precursors, CID was performed at 16 Hz and for low abundance, at 4 Hz. We used Bruker Compass DataAnalysis software 4.3 for recalibration of the data for the internal standard. MS/MS peak list was generated by ProteinScape software 3.1 and the labelled peptides were identified by Byonic 2.15.7 software. Supplementary Table 4 shows the parameters of the Byonic search engine.

### Processing of the MS results

The experiments on the nanoLC-MS/MS method identified carboxyl-labelled peptides belonging to distinct proteins. While evaluating the results of the Byonic Search Engine, we set the LogProb cut off value greater or equal to 2.00. This way, the failure rate of the results (FDR) is only 1%.

The identified peptides were searched again by blastp on the human proteome in order to identify all proteins that contained similar peptides and could not be differentiated based on nanoLC-MS/MS analysis alone. For further selection, only those proteins were considered that contained at least one peptide with modified carboxyl group and identified at least three times independently. Proteins that share peptides with significant sequence homologies were grouped into clusters. Topologies of TMPs were predicted by CCTOP algorithm. For evaluating topological localisation of labelled carboxyl groups, we used former results of experiments collected in the TOPDB database.

## Supplementary information

Supplement file

Supplement file

Supplement file

Supplement file
