# Supplementary material for: Covalently modified carboxyl side chains on cell surface leads to a novel method toward topology analysis of transmembrane proteins
Source: Sci Rep. 2019 Oct 31;9:15729. doi: 10.1038/s41598-019-52188-4 (PMC6823493; doi:10.1038/s41598-019-52188-4)
Supplement: Supplementary file 1 — Supplement file [file 41598_2019_52188_MOESM1_ESM.pdf]

Supplementary information for

Covalently modified carboxyl side chains on cell surface leads to a novel method toward topology analysis of transmembrane proteins

Anna Müller[1], Tamás Langó[1], Lilla Turiák[2], András Ács[2], György Várady[1], Nóra Kucsma[1], László Drahos[2] & Gábor E. Tusnády[1]*

[1] Institute of Enzymology, RCNS, Hungarian Academy of Sciences, Magyar Tudósok krt 2, Budapest, H-1117 Hungary.

[2] Institute of Organic Chemistry, RCNS, Hungarian Academy of Sciences, Magyar Tudósok krt 2, Budapest, H-1117 Hungary.

*Correspondence and requests for materials should be addressed to G.E.T. (email: tusnady.gabor@ttk.mta.hu)

Supplementary methods

Dot Blot analysis

The labelled and washed cells were lysed and pelleted as written in Membrane Preparation section. The supernatants of the 1700 g centrifugation were blotted onto a PVDF membrane using a Bio-Dot microfiltration apparatus.

PVDF membranes were blocked by a blocking buffer (pH=7.4; 25 mM TRIS-HCl, 2.7 mM KCl, 137 mM NaCl, 0.05% Tween-20) supplemented with 6% BSA for 45 minutes at room temperature. It was followed by incubation with HRP-conjugated Streptavidin (50000x diluted) for 45 minutes at room temperature also in blocking buffer. For the visualisation of the biotinylated peptides, Immobilon Western Chemiluminescent HRP Substrate was applied. All the images were captured by a ChemiDoc XRS+ Imaging system (Bio-Rad Laboratories, Hercules, CA, USA).

Supplementary figures

Supplementary Figure 1. The ionisation states of Biotinyl Cystamine depending on pH

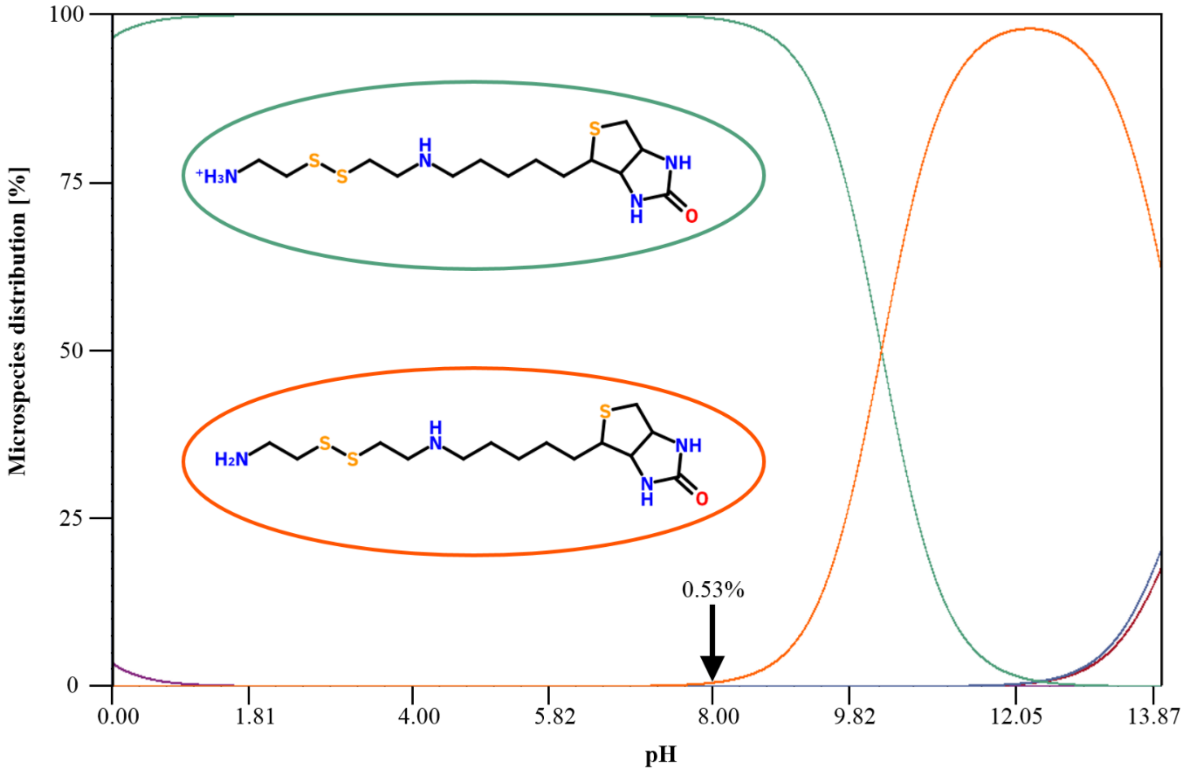

Supplementary Figure 1. The ionisation states of the applied biotinylating agent

The presence of protein amine residues in the extracellular region is also significant because deprotonated lysine side chains are also able to nucleophile attack any available activated carboxyl group. According to the diagram created by Marvin Sketch 19.2 (ChemAxon Ltd., Budapest, Hungary), 0.53% of the amount of the applied biotinylating agent (Biotinyl Cystamine) is deprotonated at the applied pH=8.0 and the temperature of 4°C.This is what makes the amid-forming reaction possible because the equilibrium will be shifted due to the appearance of the covalent bounds.

Supplementary Figure 2. Full-length SDS-PAGE and Western Blot pictures

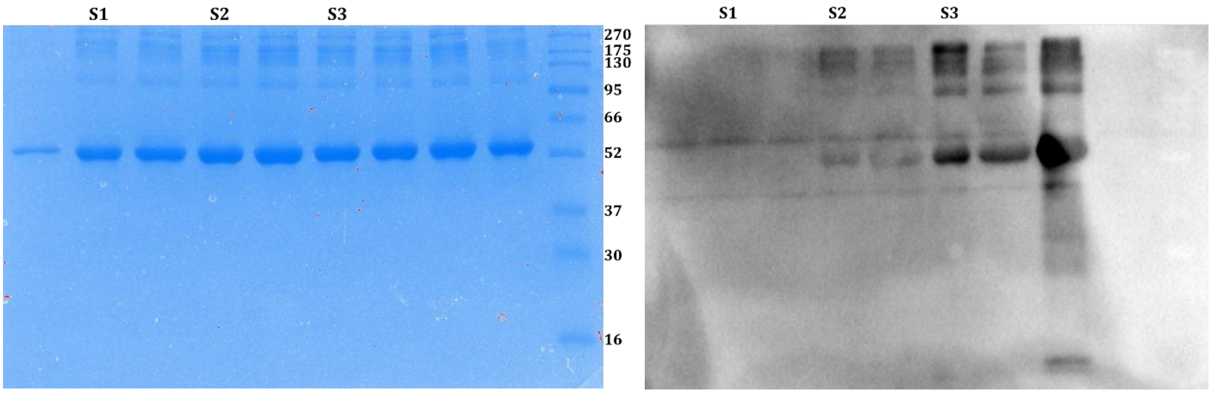

Supplementary Figure 2. Full-length SDS-PAGE and Western Blot pictures

Supplementary Figure 3. Dot Blot analysis of the labelling efficiencies

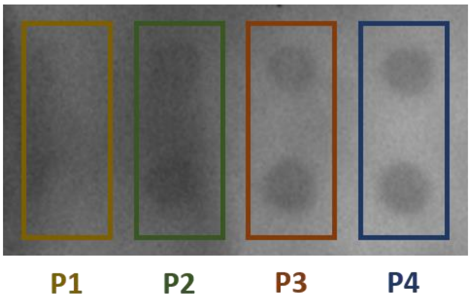

**P1**	0 mM EDC & 0 mM Sulfo-NHS
**P2**	50 mM EDC & 100 mM Sulfo-NHS
**P3**	100 mM EDC & 200 mM Sulfo-NHS
**P4**	150 mM EDC & 300 mM Sulfo-NHS

Supplementary Figure 3. Dot Blot analysis of the labelled membrane samples

In the preliminary cell experiments, we tested four different concentrations of the activating agents. As a result, we identified the highest biotinylation yield at around 50 mM EDC and 100 mM Sulfo-NHS concentrations.

Supplementary Figure 4. Flow cytometry – Propidium Iodide uptake

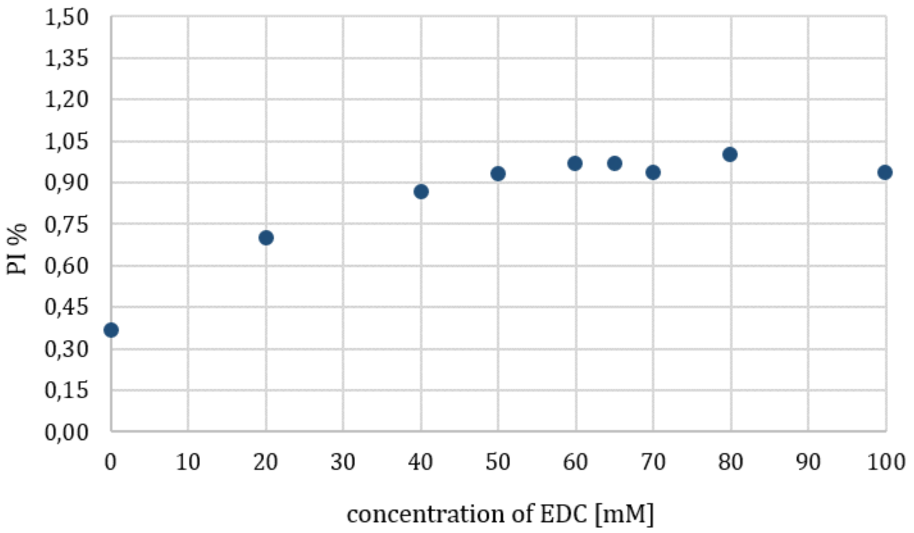

Supplementary Figure 4. The Propidium Iodide uptake of gated cells

Beside the detection of CF488A anti-biotin fluorescent antibody, it was also necessary to measure the integrity of the analysed cells. For the experiments, we applied Propidium Iodide DNA-dye that is only able to enter the intracellular region of damaged cells. Here, we present the percentage of gated cells that emitted the fluorescence of the agent.

Supplementary Figure 5. Control experiments by confocal microscopy

		Alexa Fluor 488	Hoechst 33342	DIC	Merged
-	Activating agents *EDC & Sulfo-NHS*				
-	Labelling agent *Biotinyl Cystamine*				
+	Anti-biotin antibody *CF488A*				
-	Activating agents *EDC & Sulfo-NHS*				
+	Labelling agent *Biotinyl Cystamine*				
+	Anti-biotin antibody *CF488A*				
+	Activating agents *EDC & Sulfo-NHS*				
-	Labelling agent *Biotinyl Cystamine*				
+	Anti-biotin antibody *CF488A*				

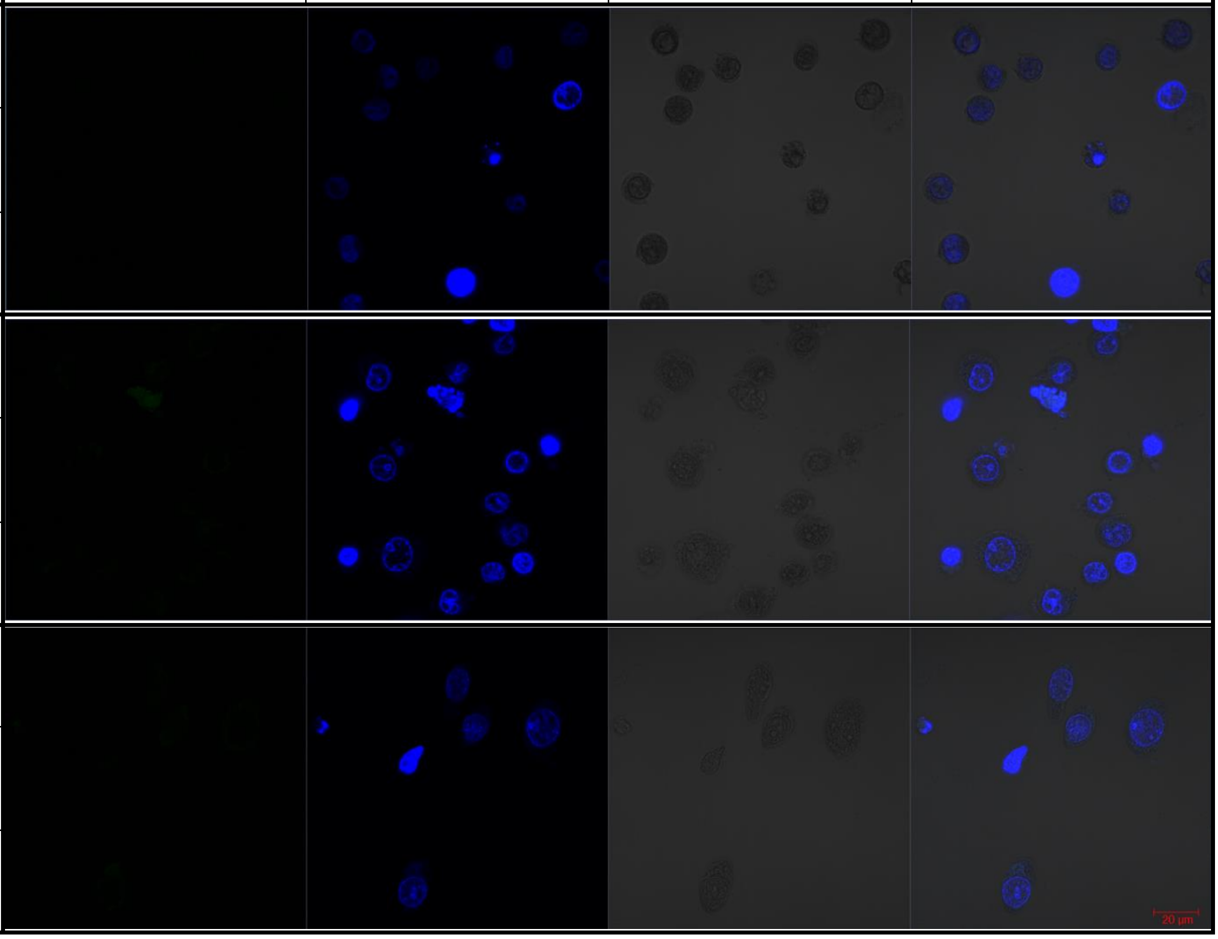

Supplementary Figure 5. Control experiments for surface protein biotinylation of HL60 cells.

Measuring the suspected maximal labelling efficiency required confocal microscopy control experiments to prove the appearance of no background fluorescence. The first lane shows the target of the measurement (from left to right: Alexa Fluor 488 conjugated anti-biotin antibody background fluorescence, Hoechst 33342 DNA dye fluorescence, Differential Interference Contrast and Merged picture). The HL60 cells were treated with different reagents, the first column indicates the applied agents for each sample („+” with or „-” without the reagent). Scale bar: 20 µm. The images were created by Zeiss ZEN lite software (Carl Zeiss, Oberkochen, Germany).

Supplementary Figure 6. Dot Blot analysis of the membrane samples and the quality of the affinity chromatography

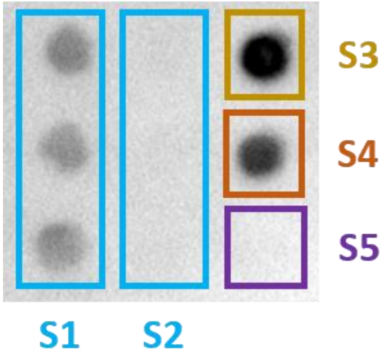

**S1**	carboxyl samples before affinity isolation
**S2**	carboxyl samples that passed through the neutravidin columns
**S3**	lysine sample of known biotin content before the tryptic digestion
**S4**	carboxyl sample before the tryptic digestion
**S5**	unlabelled sample before the tryptic digestion

Supplementary Figure 6. Dot Blot analysis of the membrane samples and the quality of the affinity chromatography

Biotin content of different membrane preparations and several fractions of the affinity chromatography were analysed by Dot Blot technique. Considering S1 and S2 dots, it is clear that the biotinylated components remained bound to the neutravidin beads after the incubation step. Admittedly, S5 dot ensures that there is no background biotin remained from original cell metabolism that would mislead the detection of the biotinylated membrane fraction.

Supplementary Figure 7. Topology predictions of Leukocyte cell surface antigen CD47

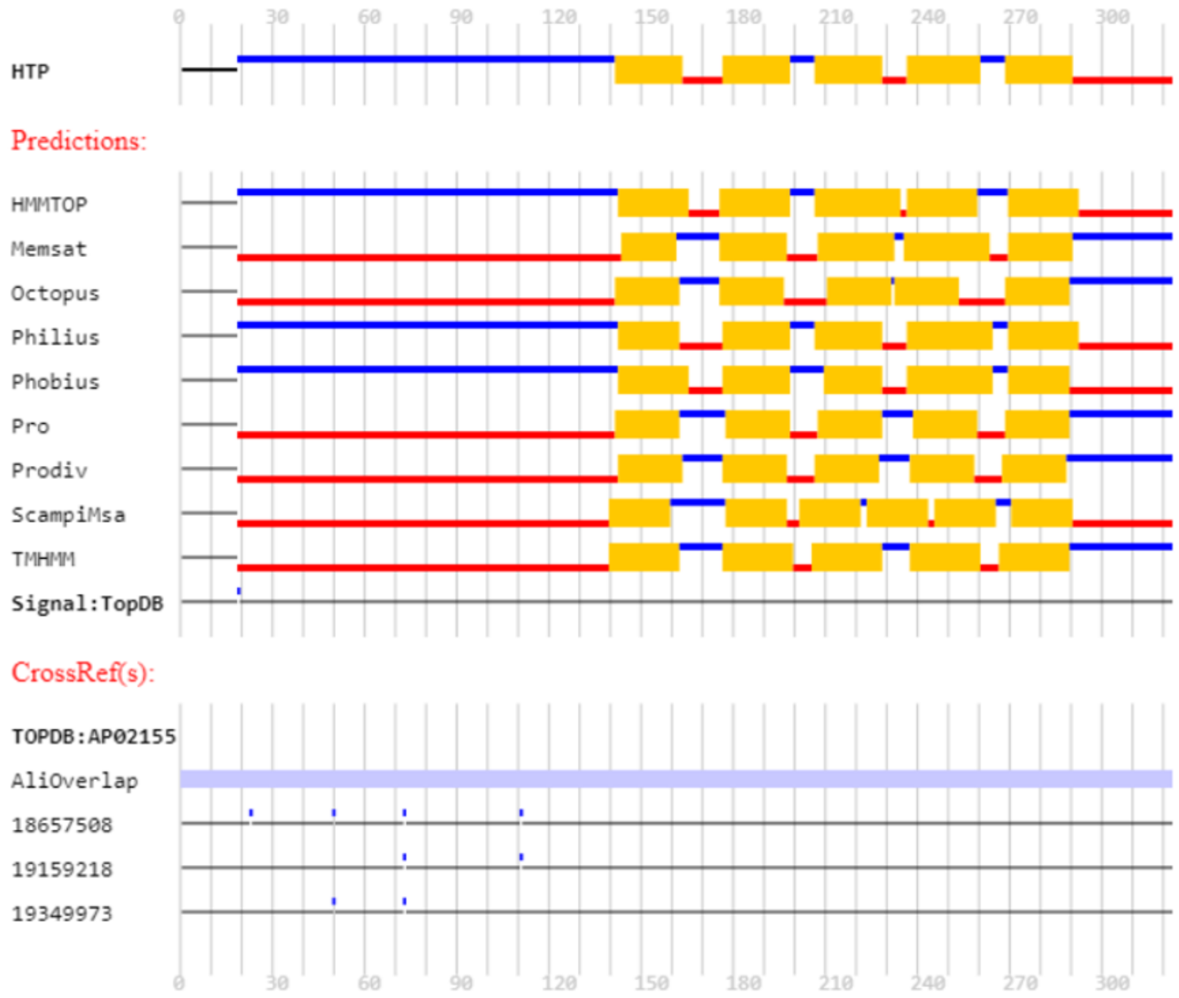

Supplementary Figure 7. Topology predictions of Leukocyte cell surface antigen CD47

According to the Human Transmembrane Proteome (HTP) database (*http://htp.enzim.hu/*), the topology predictions of Leukocyte cell surface antigen CD47 protein are quite controversial although four extracellular amino acid have already been determined in the first region. Blue lines belong to the extracellular region and red lines to the intracellular. Thicker yellow units represent the membrane region.

Supplementary Figure 8. Solved Structure for AAAT_HUMAN protein by PyMOL

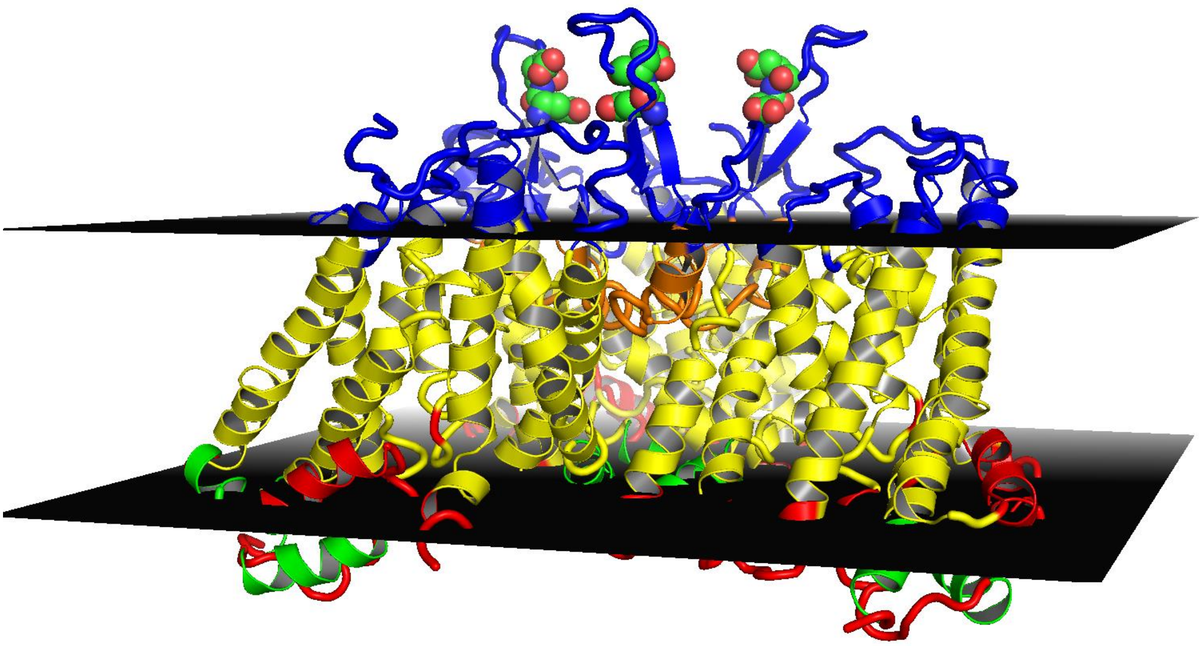

Supplementary Figure 8. Solved Structure for AAAT_HUMAN protein by PyMOL

The 3D structure of the trimer Neutral amino acid transporter B(0) protein (PDB: 6GCT) was solved previously by cryo-electron microscopy. The structure of the protein is represented by using PyMOL software (version 1.8.4.0 Open-Source, https://pymol.org/). The colours are based on topology (blue: extracellular region; yellow: membrane region; orange: re-entrant loop; red: cytosolic region; black sheets: borders of the membrane). The captured extracellular amino acids are marked with green-red spheres.

Supplementary Tables

Supplementary Table 1. List of the labelled BSA peptides

see Supplementary_Table_1.xlsx

Supplementary Table 2. List of the labelled HL60 peptides and TMP positions

see Supplementary_Table_2.xlsx

Supplementary Table 3. List of the predicted topology of the modified TMPs including the modified aspartic and glutamic acids from our experiments and already existing other experimental results

see Supplementary_Table_3.pdf

Supplementary Table 4. Byonic Search Engine parameters

**Protein database**	Swissprot Homo Sapiens
**Spectrum-level FDR**	Auto cut
**Cleavage residues**	RK
**Digest cutter**	C-terminal cutter
**Peptide termini**	Semi specific N-ragged
**Maximum number of missed cleavages**	2
**Fragmentation type**	CID low energy
**Precursor tolerance**	10.0 ppm
**Fragment tolerance**	20.0 ppm
**Charges applied to charge-unassigned spectra**	1,2,3
**Precursor mass max**	10000.0
**N-glycan search**	None
**O-glycan search**	None
**Off by x isotopes**	-2,-1,0,+1,+2
**Contaminants added**	false
**Decoys added**	true
**Disulfide Enable**	false
**Trisulfide Enable**	false
**DSS Crosslink Enable**	false
**Custom Crosslink Enable**	false
**Wildcard Enable**	0
**Combyne cut off score**	Auto
**Protein FDR cutoff**	1%
**Focused DB created**	false
**Export mzIdentML**	false
**Score version**	2
**precursor assignment flags**	2
**po NumberMonosReturn**	2
**Lock mass list**	None
**common modifications max**	1
**rare modifications max**	1

Supplementary Table 5. The allowed covalent modifications in the Byonic Search Engine

Name	Mass shift (Da)	Target amino acid or protein terminal	Structure
**Carbamidomethyl**	**+57.021464**	**Cysteine (C)**	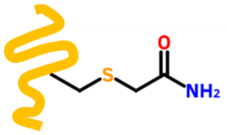
**Custom**	**+59.019355**	**Aspartic acid (E), Glutamic acid (D), Protein C-terminal**	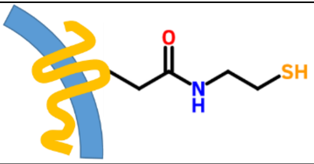
**Custom**	**+116.040819**	**Aspartic acid (E), Glutamic acid (D), Protein C-terminal**	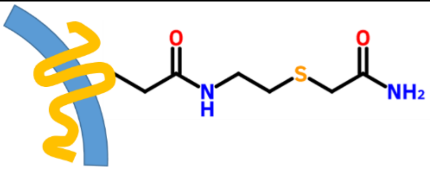

Supplementary Table 5. The allowed covalent modifications in the Byonic Search Engine

Based on the applied experimental parameters, there are three covalent modifications that we are directly searching for in the MS results. Carbamidomethyl modifications of +57.021464 Da can occur on the Cysteine side chains in every region (both extracellular and intracellular) of the whole protein because Iodoacetamide alkylating agent is applied in several phases of the protocol (also after the isolation of TMPs). The Custom modifications of +59.019355 and +116.040819 Da appear only in the extracellular region of the TMPs because we modify the carboxyl groups of the intact cell surface at the beginning of the protocol. Here we also present the structure of these modifications completed with their target location in native proteins (Yellow curve: protein structure, Blue arch: membrane).

Supplementary Table 6. Additional modifications in the Byonic Search Engine

Name	Mass shift (Da)	Location
Dioxidation	+31.989829	Triptophan
Deamidated	+0.984016	Asparagine
Deamidated	+0.984016	Glutamine
Gln → pyro-Glu	-17.026549	Glutamine N-terminal
Glu → pyro-Glu	-18.010565	Glutamic acid N-terminal
Ammonia-loss	-17.026549	Cysteine N-terminal
Acetyl	+42.010565	Protein N-terminal

Supplementary Table 7. List of reagents and their suppliers

Name	Producer
Ammonium bicarbonate	Sigma-Aldrich
Anti-Biotin-CF Dye Conjugate CF488A	Sigma-Aldrich
Biotinyl Cystamine	Santa Cruz Biotechnology
Bovine Serum Albumin	Sigma-Aldrich
Coomassie Brilliant Blue CBB R-250	Bio-Rad
Disodium phosphate	Sigma-Aldrich
1,4-dithiothreitol	Thermo Scientific
1-ethyl-3-(3-dimethylaminopropyl) carbodiimide hydrochloride	Thermo Scientific
Fetal Bovine Serum	Thermo Scientific
Glycine	Sigma-Aldrich
Hoechst 33342 DNA-dye	Molecular Probes
HRP-conjugated Streptavidin	Merck
Immobilon Western Chemiluminescent HRP Substrate	Millipore
Iodoacetamide	Sigma-Aldrich
Monopotassium phosphate	Sigma-Aldrich
2-(4-morpholino) ethanesulfonic acid hydrate	Sigma-Aldrich
Na-tosyl-L-lysine chloromethyl ketone hydrochloride (TLCK)	Sigma-Aldrich
α2-3,6,8,9 Neuraminidase A	New England Biolabs
Neutravidin agarose beads	Thermo Scientific
N-hydroxysulfosuccinimide (Sulfo-NHS)	Sigma-Aldrich
Penicillin-Streptomycin Solution	Thermo Scientific
PNGaseF	New England Biolabs
Potassium chloride	Sigma-Aldrich
Propidium Iodide	Thermo Scientific
Rapigest	Waters
RPMI 1640 medium	Thermo Scientific
Sodium bicarbonate	Sigma-Aldrich
Sodium chloride	Sigma-Aldrich
Sodium dodecyl sulfate (SDS)	Sigma-Aldrich
Sucrose	Sigma-Aldrich
2,2′ -thiodiethanol	Sigma-Aldrich
Trypsin (MS-grade)	Sigma-Aldrich
Tris hydrochloride	Bio-Rad

Supplementary Table 8. List of applied instruments and machines

Name	Producer	Additional information
Bio-Dot Microfiltration System	Bio-Rad	
Bruker Maxis II ETD Q-TOF mass spectrometer	Bruker	
C18 spin column	Thermo Scientific	
Cell counting chamber	Hirschmann Instruments	
Centrifuge	Eppendorf	5810 R
ChemiDoc XRS+	Bio-Rad	
Confocal Microscope	Zeiss	LSCM 710
Dionex Ultimate 3000 NanoLC System	Thermo Scientific	
Elmasonic S 30 (H) Ultrasonic cleaning unit	Elma Schmidbauer GmbH	
FACS Attune Acoustic Focusing Cytometer	Applied Biosystem	
Filtropur vacuum filter	Sarstedt	V50, 500ml, 0.2µm
26-gauge needle	Sigma	
Incubator	Eppendorf	Galaxy 170R
Objective for Confocal Microscope	Zeiss	63x NA=1.4 Plan Apo
Polycarbonate UC tube	Beckman	10.4 ml
PowerPac Universal	Bio-Rad	
Streamline Class II Biological Safety Cabinet	Esco	
Synergy Mx Microplate Reader	BioTek	
T25, T75 flask	Eppendorf	
Thermoblock	Biosan	CH-100
UC fixed rotor	Beckman	70.1 Ti
Ultracentrifuge	Beckman	L7-55
