# Supplementary material for: Covalently modified carboxyl side chains on cell surface leads to a novel method toward topology analysis of transmembrane proteins
Source: Sci Rep. 2019 Oct 31;9:15729. doi: 10.1038/s41598-019-52188-4 (PMC6823493; doi:10.1038/s41598-019-52188-4)
Supplement: Supplementary file 2 — Supplement file [file 41598_2019_52188_MOESM2_ESM.pdf]

Supplementary Table 3.

List of the predicted topology of the modified TMPs including the modified aspartic and glutamic acids from our experiments and already existing other experimental results

Legend of the graphics:

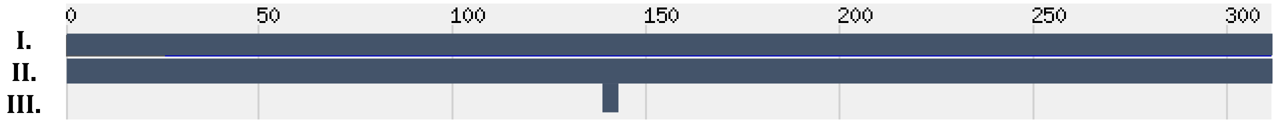

I. line: CCTOP Prediction
II. Line: Previous other experimental results*
III. Line: Identified glutamic or aspartic acid

*Continuous blue or red line means that the structure of the whole region of the protein was previously determined.

Legend of the colours used in topology representation:

Grey: signal sequence
Blue: extracellular region
Yellow: membrane region
Red: intracellular region

UNIPROT ID **Topology representation**

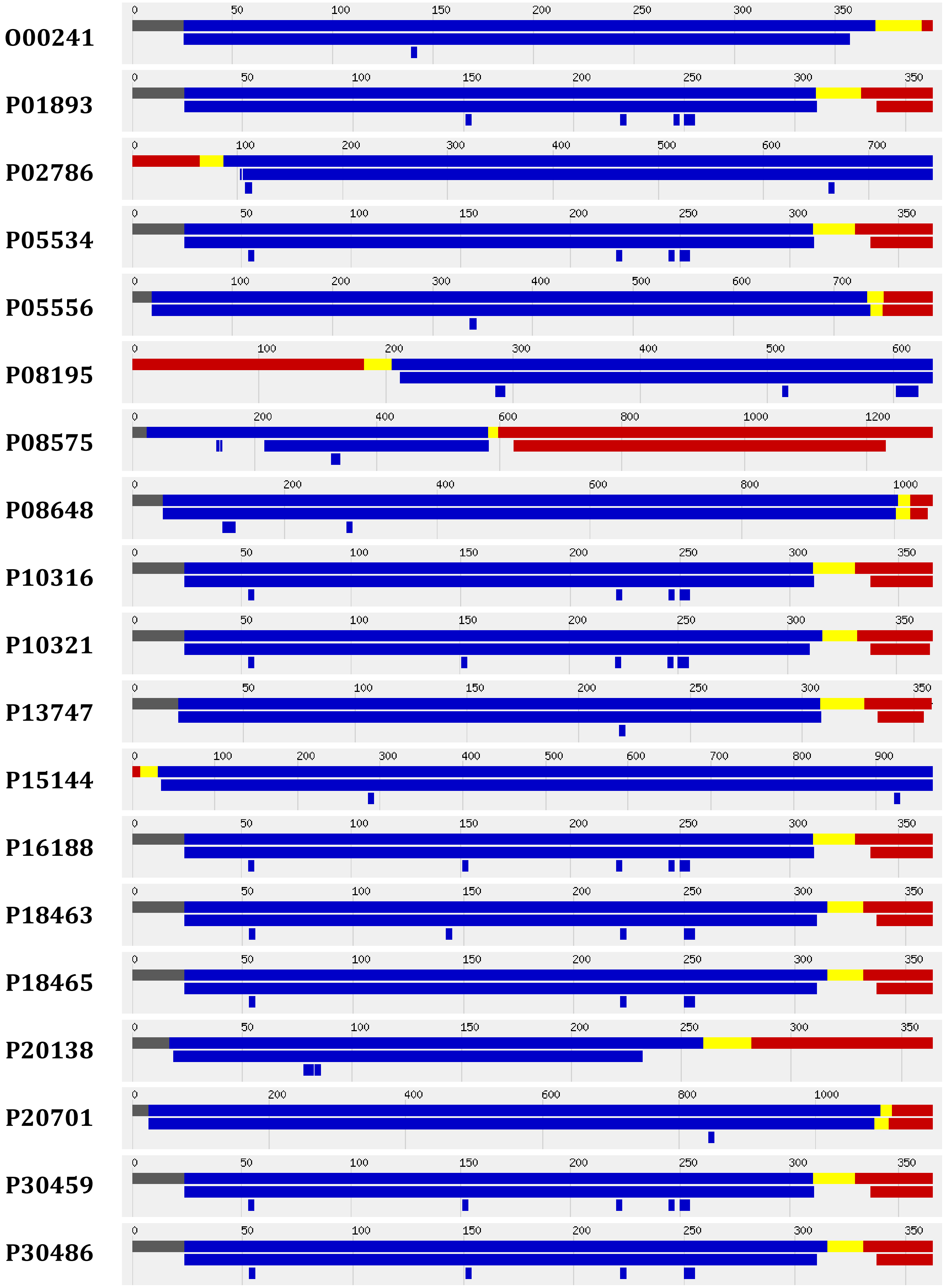

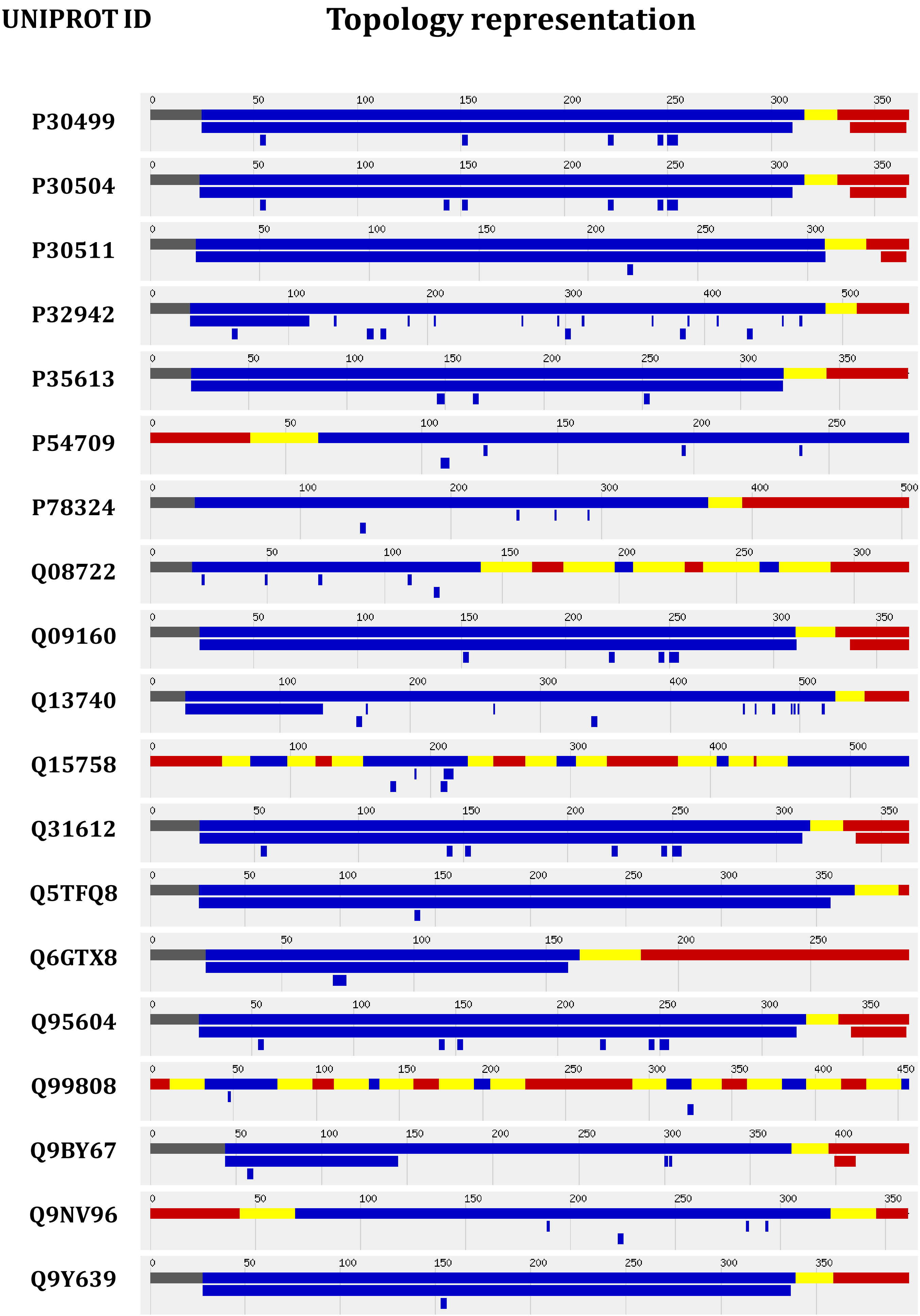
UNIPROT ID
Topology representation
P30499
P30504
P30511
P32942
P35613
P54709
P78324
Q08722
Q09160
Q13740
Q15758
Q31612
Q5TFQ8
Q6GTX8
Q95604
Q99808
Q9BY67
Q9NV96
Q9Y639
